# Epidemiology and characterization of *Providencia stuartii* isolated from hospitalized patients in southern Brazil: a possible emerging pathogen

**DOI:** 10.1099/acmi.0.000652.v4

**Published:** 2023-10-18

**Authors:** Gustavo Henrique Migliorini Guidone, Jennifer Germiniani Cardozo, Luana Carvalho Silva, Matheus Silva Sanches, Ligia Carla Faccin Galhardi, Renata Katsuko Takayama Kobayashi, Eliana Carolina Vespero, Sergio Paulo Dejato Rocha

**Affiliations:** ^1^​ Laboratory of Bacteriology, Department of Microbiology, Center of Biological Sciences, State University of Londrina, Londrina, Brazil; ^2^​ Laboratory of Basic and Applied Bacteriology, Department of Microbiology, Center of Biological Sciences, State University of Londrina, Londrina, Brazil; ^3^​ Virology Laboratory, Department of Microbiology, Center of Biological Sciences, State University of Londrina, Londrina, Brazil; ^4^​ Department of Pathology, Clinical and Toxicological Analysis, Health Sciences Center, University Hospital of Londrina, State University of Londrina, Paraná, Brazil

**Keywords:** hospital infection, virulence factors, pathogenicity, emergence pathogen

## Abstract

This study aimed to characterize the virulence factors and antimicrobial resistance of *Providencia stuartii *, an opportunistic pathogen that causes human infections. We examined 45 isolates of *P. stuartii* both genotypically and phenotypically by studying their adherence to HeLa cells, biofilm formation, cytotoxicity and antimicrobial resistance, and analysed their genomes for putative virulence and resistance genes. This study found that most isolates possessed multiple virulence genes, including *fim*A, *mrk*A, *fpt*A, *iut*A, *ire*A and *hly*A, and were cytotoxic to Vero cells. All the isolates were resistant to amoxicillin plus clavulanic acid, levofloxacin and sulfamethoxazole plus trimethoprim, and most were resistant to ceftriaxone and cefepime. All isolates harboured extended-spectrum beta-lactamase coding genes such as *bla*_CTX-M-2_ and 23/45(51.11 %) of them also harboured *bla*_CTX-M-9_. The gene KPC-2 (carbapenemase) was detected in 8/45(17.77 %) isolates. This study also found clonality among the isolates, indicating the possible spread of the pathogen among patients at the hospital. These results have significant clinical and epidemiological implications and emphasize the importance of a continued understanding of the virulence and antimicrobial resistance of this pathogen for the prevention and treatment of future infections.

## Data Summary

No data was generated during this research

## Introduction

*Providencia* is a genus composed of ubiquitous facultative anaerobic micro-organisms that are mobile through the use of peritrichous flagella [1]. Currently, the genus comprises nine species, two of which, *Providencia stuartii* and *Providencia rettgeri *, are most commonly associated with infections in humans [2].

The incidence of *P. stuartii *, which is associated with infections in hospitalized patients, is approximately 4 per 100 000; however, the incidence of *P. stuartii* in urinary infections is 9 % [3, 4]. In addition to urinary infections, *P. stuartii* can cause conjunctivitis, endocarditis, meningitis and suprahepatic abscesses [5–8].

*P. stuartii* is intrinsically resistant to several commonly used antimicrobials such as first-generation cephalosporins, polymyxins and aminopenicillins. Therefore, treatment is often limited to carbapenems, aminoglycosides and quinolones, and reports of resistance to these agents are increasing frequent [9].

In addition to antimicrobial resistance, pathogens possess other characteristics that enable infection. The ability of pathogenic bacteria to cause diseases in susceptible hosts is facilitated by the combined action of several virulence factors during infection [10].

To date, the prevalence of virulence-related genes in clinical strains of *P. stuartii* has not been reported, and further studies are needed to determine the prevalent virulence genes in this species. This study aimed to genotypically and phenotypically characterize the virulence factors and antimicrobial resistance of *P. stuartii* isolates from different human infections.

## Methods

### Bacterial sampling

The 45 *P *. *stuartii* strains analysed in this study were isolated from patients hospitalized at the Regional University Hospital of Northern Paraná, Brazil. Isolates were selected for being multidrug-resistant (MDR) according to previously described criteria [11]. This classifies multidrug-resistant bacteria as bacteria not susceptible to at least one agent in three or more categories of antimicrobials, using criteria determined by the Clinical Laboratory Standards Institute (CLSI), European Committee for Antimicrobial Susceptibility Testing (EUCAST), or Food and Drug Administration (FDA) of the United States.

The isolates were collected between February 2014 and December 2017. The identification and the antibiogram were determined using the Vitek 2 Compact system (BioMérieux, Marcy-l’Etoile, France). The antibiogram was confirmed using a disc-diffusion test (Kirby–Bauer) according to the CLSI. Samples were tested for amoxicillin plus clavulanic acid, levofloxacin, sulfamethoxazole plus trimethoprim, ceftriaxone, imipenem, cefepime, ertapenem, meropenem, aztreonam and ceftazidime.

This study was approved by the Research Ethics Committee of the State University of Londrina (CEP-1.590.120). This study evaluated 45 bacterial isolates obtained from 34 patients (23 men and 11 women) admitted to our hospital. The patients were aged 24–85 years (mean of 54.67 years).

### *In silico* determination of virulence genes

The sequenced genome of *P. stuartii* ATCC 33672 (GenBank accession number CP008920) was analysed using the VFanalyzer tool on the Virulence Factor of Pathogenic Bacteria (VFDB) platform (available at http://www.mgc.ac.cn/cgi-bin/VFs/v5/main.cgi?func=VFanalyzer). The analysis revealed a high sequence identity with previously described virulence genes. The genome of *P. stuartii* ATCC 33672 was compared to those of *Escherichia coli* UPEC CFT073 (GenBank NC_004431) and *Klebsiella pneumoniae* KCTC 2242 (GenBank GCA_000220485.1) strains. The comparison was performed with known enterobacteria, with the aim of verifying possible homologous genes between these pathogens.

### Detection of virulence genes

Bacterial DNA was extracted using the boiling method, followed by heat shock. In brief, the isolates were cultured in Luria–Bertani broth for 24 h at 37 °C. Following incubation, the cell culture was transferred to 1.5 ml microtubes and centrifuged at 10 000 r.p.m. for 10 min. After discarding the supernatant, the pellet containing cellular material was resuspended in 300 µl of sterilized ultrapure water. The microtubes were then boiled for 10 min, followed by a 5 min cooling period on ice. Subsequently, the microtubes were centrifuged at 10 000 r.p.m. for 5 min and the resulting bacterial lysate containing the DNA was carefully transferred to another sterile microtube.

The oligonucleotide primers are listed in Table 1. Primers were designed using the PrimerQuest Tool available at (idtdna.com/PrimerQuest/Home/Index). PCR was performed using a GeneAmp PCR System 9700 thermocycler (Applied Biosystems) in a final volume of 25 µl containing 1.0 µl of MgCl_2_ (2 mM, Invitrogen), 2.5 of 10×buffer (Invitrogen), 2.5 µl of dNTPs (10 mM; Invitrogen), 0.5 µl (0.5 mM) of forward and reverse oligonucleotides (Invitrogen), 0.5 µl (1 U) of Taq DNA polymerase (Invitrogen), 2.0 µl of bacterial lysate, and 15.5 µl of ultrapure water (Millipore). The PCR products were separated by electrophoresis on a 1 % agarose gel and stained with SYBR Safe (Invitrogen) dissolved in TBE buffer (89 mM Tris base, 89 mM boric acid, and 2 mM EDTA, pH 8.3). A 1 kb Ladder (Invitrogen) was used as a molecular marker, and the amplicons were visualized using a UV light transilluminator (Vilbert Loumart).

**Table 1.** Oligonucleotide primers of virulence-related genes of *P. stuartii *.

Genes		Oligonucleotide sequence	Fragment length	Temp	Reference
Adhesins	*fim*A	(F) GCATGTGCTGTTGATGCTAAC	215 pb	55 °C	This study
		(R) GAACCCGCAACAGCTAGAA			
	*mrk*A	(F) GCCAAGGTAGTGACGCTAAA	411 pb	55 °C	This study
		(R) TTTCTACGATACCGCTGCTAAC			
Siderophores	*fpt*A	(F) ACTGCTCCATGGGCTTTATC	744 pb	55 °C	This study
		(R) CATAGGTACGCCAGCCATATT			
	*ire*A	(F) CCCGGCGAATACACCTTAAT	351 pb	55 °C	This study
		(R) GTAATCGCCACCACCATACA			
	*iut*A	(F) TCTGGCGCGACTTCTTTATATG	347 pb	55 °C	This study
		(R) GTGGCTTGTAGCTGCTGATTA			
Hemolysin	*hly*A	(F) TCGCCATCTTAGCAGGTATTG	701 pb	55 °C	This study
		(R) AGCTTCGAGTGTATCGGAAAG			

F, forward; Pb, base pairs; R, reverse; Temp, Annealing temperature.

### Detection of resistance genes

Resistance genes were detected by PCR as described in Section 2.3. All isolates were analysed for the presence of *bla*_CTX-M_ types 1, 2, 8, 9, and 25 [12], KPC-2 [13] and NDM [14].

### HeLa cell-adhesion assay

Cell adhesion was evaluated using a previously described technique [15]. The interaction between the cells and bacteria was tested for 6 h. HeLa cells were grown on 13 mm coverslips in 24-well plates containing 1 ml of Dulbecco’s modified Eagle’s medium (DMEM; Difco). After a cell monolayer was formed, the medium was aspirated and the wells were washed three times with PBS (Na_2_HPO_4_ 9.7 mM, KH_2_PO_4_ 1.25 mM, NaCl 137.93 mM, and KCl 2.68 mM). Next, 1 ml of DMEM supplemented with FBS (Difco) and 1 % d-mannose was added to each well, and a 40 µl aliquot of bacterial culture grown in tryptic soy broth (TSB; Difco) was added to each well. The plates were incubated for 3 h at 37 °C, and then the wells were washed three times with 1 ml of sterile PBS to remove non-adherent bacteria. Then, DMEM containing FBS and 1 % d-mannose was added to each well, and the plates were incubated for 3 h at 37 °C. After a 6 h incubation, the wells were washed three times with PBS, and the cells on the coverslips were fixed with 100 % methanol and stained with May−Grünwald stain for 5 min, followed by Giemsa stain for 20 min. The coverslips were observed under a light microscope at 1000×magnification. HeLa cells were used as the negative controls. As positive controls, we used *E. coli* strain E2348/69 for localized adhesion [16], *E. coli* C1845 for diffuse adhesion [17] and *E. coli* 042 for aggregative-type adhesion [18].

### Biofilm formation assay

All isolates were subjected to a biofilm formation assay performed in polystyrene 96-well plates using crystal violet as described previously [19]. *E. coli* 042 [18] was used as the positive control, and TSB (Difco) was used as the negative control. Absorbance (A) was measured at 570 nm using a spectrophotometer, and biofilm formation was classified as absent, weak, moderate, strong or very strong [19].

### Haemolytic activity assay

Haemolytic activity on blood agar containing 5 % sheep blood was evaluated using a previously described method [20]. To prepare the blood agar plates, freshly sterilized and cooled nutrient agar medium (45–50 °C) was aseptically supplemented with 5 % sheep blood. The test isolates were then inoculated on the blood agar plates, which were incubated overnight at 37 °C. After incubation, the haemolytic activity of the isolates was examined, and the isolates that produced a clear zone were considered haemolytic.

### Assay for cytotoxicity against Vero cells

The cytotoxicity of all isolates was assessed against Vero cells (African green monkey kidney) as described previously [21]. All isolates were cultivated in 3 ml of TSB (Difco) for 18 h at 37 °C with shaking at 180 r.p.m. The broth was then centrifuged at 13 000 ***g*** for 10 min. The supernatants were collected and filtered (47 mm PVDF syringe filters with 0.22 µm pores; Durapore). Filtered supernatants were serially diluted (1 : 10) in 96-well polystyrene plates and incubated for 72 h at 37 °C and 5 % CO_2_.

Cytotoxicity of the isolates was quantified by measuring the metabolic activity of Vero cells incubated with the bacteria supernatant using the (3-[4,5-dimethylthiazol-2-yl]−2,5-diphenyltetrazolium) MTT assay [22]. The supernatant of *E. coli* O157:H7 (EDL933) was used as a positive control for cytotoxicity [23]. Vero cells without the bacterial culture supernatant were used as negative controls. A strain was considered highly cytotoxic when cell death was ≥50 % compared to the negative control.

### Enterobacterial repetitive intergenic consensus PCR (ERIC-PCR)

The genetic similarity of the isolates was determined by ERIC-PCR analysis using primers ERIC-1R (5′-ATGTAAGCTCCTGGGGATTCAC-3′) and ERIC-2 (5′-AAGTAAGTGACTGGGGTGAGCG-3′) as previously described [24].

Each 25 µl reaction contained 2 mM MgCl_2_ (Invitrogen), 10×buffer (Invitrogen), 1.25 mM dNTPs (Invitrogen), 50 pmol of forward primer, 50 pmol of reverse primer (Invitrogen), 2 U of Taq DNA polymerase (Invitrogen) and 100 ng of DNA template. For DNA amplification, the cycling conditions consisted of an initial denaturation step of 5 min at 94 °C, followed by 40 cycles of denaturation for 1 min at 94 °C, primer annealing for 1 min at 50 °C, and extension for 5 min at 72 °C, and a final extension step at 72 °C for 10 min. The PCR products were subjected to 1.5 % agarose gel electrophoresis for 4 h in TBE buffer, stained with ethidium bromide (0.5 µg ml^−1^), and visualized using a UV transilluminator (Vilbert Loumart).

A similarity dendrogram was constructed using the Gel J 2.0 software [25], unweighted pair group method with arithmetic mean (UPGMA), and data similarity coefficient for cluster analysis (Jaccard). The standard cutoff for defining a cluster was 85 %, which was the standard cutoff for Gram-negative bacteria used for epidemiological purposes in a previous study [26].

## Results

### Virulence genotypes of the isolates

Genome analysis of *P. stuartii* ATCC 33672 revealed several putative genes with high sequence identity to those encoding common virulence factors in other enterobacteria (Tables S1 and S2, available in the online version of this article). Six of these were selected for analysis of the clinical isolates: two fimbriae genes (*fim*A and *mrk*A), three siderophore receptors (*ire*A, *fpt*A and *iut*A), and haemolysin (*hly*A) (Fig. S1). All isolates analysed had virulence-related genes that may contribute to their pathogenesis in humans. The *fim*A, *mrk*A, *fpt*A, *iut*A and *hly*A genes were present in all analysed isolates, and the *ire*A gene was found in 93.33 % of isolates.

### Virulence phenotypes of the isolates

**Fig. 1.**
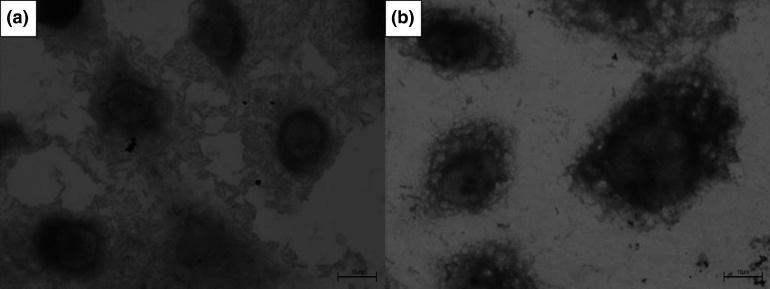
Adhesion of *P. stuartii* in HeLa cells. (**a**) Adhesion of *P. stuartii* in HeLa cells. (**b**) Bacterial cells not adhered by inhibition of type 1 fimbriae by d-mannose treatment.

Phenotypic characterization showed that all isolates adhered to HeLa cells without d-mannose supplementation. However, in the presence of d-mannose supplementation, only 18/45 isolates (40 %) adhered to HeLa cells, whereas the remaining 27/45(60 %) did not (Fig. 1).

All the isolates formed biofilms. However, the extent of biofilm formation varied. Among the isolates, (10/45) 22.22 %, (22/45) 48.89 % and (13/45) 28.89 % were strong, moderate and weak biofilm formers, respectively (Fig. S2).

The cytotoxicity against Vero cells revealed that all isolates were cytotoxic to Vero cells, and 33/45 isolates (73.33 %) were considered highly cytotoxic, as they killed 50 % or more of the cultured cells. Haemolytic activity was investigated on blood agar, and all isolates were found to be haemolytic, showing lysis zones on blood agar after 24 h of incubation (Fig. S3).

### Antibiotic resistance of clinical isolates

**Fig. 2.**
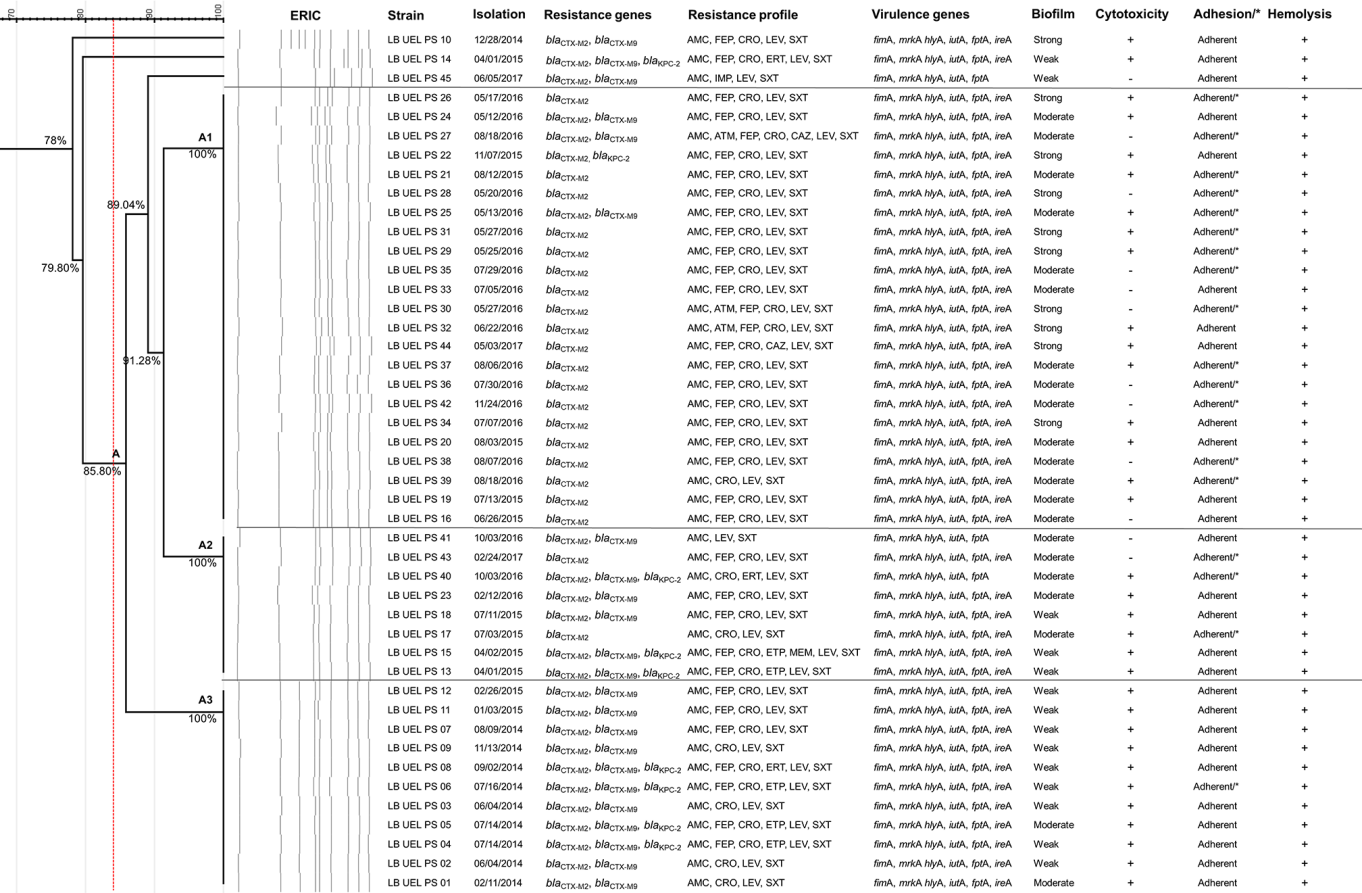
Dendrogram of genetic similarity, resistance and virulence profile of isolates of *P. stuartii *. AMC, amoxicillin plus clavulanic acid; LEV, levofloxacin; SXT, trimetoprim plus sulfamethoxazole; CRO, ceftriaxone; IMP, imipenem; FEP, cefepime; ETP, ertapenem; MEM, meropenem; ATM, aztreonam; CAZ, ceftazidime; +, positive for the test; -, negative for the test; * Considered adherent in the adherence test with d-mannose supplementation.

All the isolates were resistant to amoxicillin plus clavulanic acid, levofloxacin and sulfamethoxazole plus trimethoprim. Among the other tested antimicrobials, 43/45(95.55 %) isolates were resistant to ceftriaxone, 36/45(80 %) to cefepime, 8/45(17.77 %) to ertapenem, 1/45(2.22 %) to meropenem, 3/45(6.66 %) to aztreonam, and 2/45(4.44 %) to ceftazidime (Fig. 2). An investigation of ESBL production, specifically *bla*_CTX-M_ genes, revealed that all isolates harboured *bla*_CTX-M-2_ genes and 23(51.11 %) isolates harboured *bla*_CTX-M-9_ genes. The *bla*_KPC-2_ gene was found in eight (17.77 %) of the analysed isolates, and the gene *bla*_NDM_ was not found.

### Genetic similarity among the isolates

The examined isolates showed 85 % similarity, and nearly all isolates, except isolates 10 and 14, grouped into one large cluster (clonal complex A). Three sub-clusters were formed with >90 % similarity (Fig. 2): sub-cluster A1 consisted of 23 isolates, sub-cluster A2 consisted of eight isolates, and sub-cluster A3 consisted of 11 isolates. One isolate, 45, did not fit into any sub-cluster. There were differences in the phenotypes, virulence and resistance genes among the isolates in each sub-cluster,

In sub-cluster A1, there was a predominance of micro-organisms isolated in 2016 (Table 2), and most isolates were positive only for *bla*_CTX-M-2_, although three isolates also harboured *bla*_CTX-M-9_. All isolates had the same virulence genes, and phenotypic characteristics varied among the isolates.

**Table 2.** Origin of the studied *P. stuartii* isolates

Patient	Isolated samples	Isolation place	Date of isolation (mm/dd/yyyy)
1	LB UEL PS 01	Urine	02/11/2014
2	LB UEL PS 02 LB UEL PS 06	Urine Urine	06/04/2014 07/16/2014
3	LB UEL PS 03	Blood	04/06/2014
4	LB UEL PS 04 LB UEL PS 08 LB UEL PS 23	Urine Secretion Bone fragment	07/14/2014 09/02/2014 02/12/2016
5	LB UEL PS 05	Urine	07/14/2014
6	LB UEL PS 07	Tracheal secretion	08/09/2014
7	LB UEL PS 09	Blood	11/13/2014
8	LB UEL PS 10 LB UEL PS 11	Blood Blood	12/28/2014 01/03/2015
9	LB UEL PS 12 LB UEL PS 14	Blood Urine	02/26/2015 04/01/2015
10	LB UEL PS 13 LB UEL PS 15	Blood Catheter	04/01/2015 04/02/2015
11	LB UEL PS 16	Urine	06/26/2015
12	LB UEL PS 17 LB UEL PS 43	Tissue Tissue	07/03/2015 02/24/2017
13	LB UEL PS 18	Urine	07/11/2015
14	LB UEL PS 19	Urine	07/13/2015
15	LB UEL PS 20	Urine	08/03/2015
16	LB UEL PS 21	Tracheal secretion	08/12/2015
17	LB UEL PS 22	Blood	11/07/2015
18	LB UEL PS 24 LB UEL PS 27	Urine Urine	05/12/2016 08/18/2016
19	LB UEL PS 25	Urine	05/13/2016
20	LB UEL PS 26	Urine	05/17/2016
21	LB UEL PS 28	Tracheal secretion	05/20/2016
22	LB UEL PS 29	Urine	05/25/2016
23	LB UEL PS 30	Urine	05/27/2016
24	LB UEL PS 31	Tracheal secretion	05/27/2016
25	LB UEL PS 32	Tracheal secretion	06/22/2016
26	LB UEL PS 33	Tracheal secretion	07/05/2016
27	LB UEL PS 34	Tracheal secretion	07/07/2016
28	LB UEL PS 35 LB UEL PS 37 LB UEL PS 39	Blood Urine Tracheal secretion	07/29/2016 08/06/2016 08/18/2016
29	LB UEL PS 36	Urine	07/30/2016
30	LB UEL PS 38	Urine	08/07/2016
31	LB UEL PS 40 LB UEL PS 41	Blood Catheter	10/03/2016 10/03/2016
32	LB UEL PS 42	Urine	11/24/2016
33	LB UEL PS 44	Urine	05/03/2017
34	LB UEL PS 45	Eyeball swab	06/05/2017

In sub-cluster A2, only two isolates lacked the *bla*_CTX-M-9_ gene. Two isolates lacked *ire*A, which encodes a siderophore receptor. The phenotypic characteristics varied among the isolates.

In sub-cluster A3, there was a predominance of micro-organisms isolated in 2014, and all isolates harboured the beta-lactamase-encoding genes *bla*_CTX-M-2_ and *bla*_CTX-M-9_, as well as all the virulence-related genes studied, and their phenotypic characteristics were similar.

Several isolates were derived from the same patient: isolates 2 and 6 were from patient 2; isolates 13 and 15 were from patient 10; isolates 17 and 43 were from patient 12; isolates 24 and 27 were from patient 18; isolates 35, 37 and 39 were from patient 28; and isolates 40 and 41 were from patient 31 (Table 2). However, these strains were isolated on different dates or from different origins, indicating that these patients had multiple infections with clonal isolates of *P. stuartii *. Interestingly, several isolates from the same patient, that is, isolates 10 and 11 (patient 8), isolates 12 and 14 (patient 9), and isolates 4, 8 and 23 (patient 4), were not grouped in the same sub-clusters despite being from the same patient, indicating that these patients were infected with different micro-organisms.

## Discussion

This study aimed to identify virulence-related genes in clinical isolates of *P. stuartii* and to associate them with their pathogenicity phenotypes. Comparison of the genome of *P. stuartii* ATCC 33672 with those of other enterobacteria revealed genes in *P. stuartii* with high identity to virulence-related genes common to these micro-organisms. Of the identified genes, six were chosen for analysis of the studied isolates: fimbriae- (*fim*A and *mrk*A), siderophore receptor- (*ire*A, *pft*A, and *iut*A) and hemolysin- (*hly*A) encoding genes.

The *fim*A gene encodes the largest subunit of type 1 fimbriae, which are involved in host-cell adhesion and colonization [27]. All the 45 isolates tested positive for *fim*A. In a study by Daga *et al*. [28], the prevalence of type 1 fimbria-encoding genes in analysed *E. coli* isolates was 95.8 %. In another study of *E. coli* isolates by Kakian *et al*. [29], the frequency of *fim*A was 74 % in isolates from hospital inpatients and 76 % in isolates from outpatients. In a study by Ghasemian *et al*. [30], the prevalence of *fim*A in *Klebsiella oxytoca* was 60 %. These data indicate a high prevalence of type 1 fimbria-encoding genes in enterobacteria.

The *mrk*A gene encodes a subunit of type 3 fimbriae. These fimbriae mediate the attachment to host epithelial cells and play important roles in biofilm formation on abiotic surfaces [31]. Ghasemian *et al*. [30] showed the *mrk*A gene was present in 42 % of *K. oxytoca* isolates.

Iron is an essential element for bacterial growth. Bacteria acquire iron by secreting siderophores that bind to iron present in their hosts. Invasive *E. coli* strains have high-affinity iron acquisition systems that compete with the siderophores of the host and favour bacterial growth when the iron content is low. *Ire*A, *iut*A and *fpt*A encode receptors that bind iron-chelating siderophores [32]. In the present study, *iut*A and *fpt*A were detected in all isolates, whereas *ire*A was detected in 93.33 % of the analysed micro-organisms.

In their study on *P. mirabilis *, Sanches *et al*. [33] detected *ire*A in all studied isolates, demonstrating its high prevalence, similar to that observed in the present study. In a study by Daga *et al*. [28], *iut*A was observed in 64.3 % of *E. coli* isolates. Kadry *et al*. [34] detected the pyochelin siderophore receptor-encoding gene *fpt*A in 85.7 % of evaluated *Pseudomonas aeruginosa* isolates, indicating a high prevalence of this gene and corroborating the findings of the present study.

The *hly*A gene encodes a haemolysin that lyses red blood cells. Despite being known for its haemolytic function, it also has cytotoxic activity against a wide range of cell types and is therefore considered an important virulence factor [35].

Type 1 fimbriae, such as those encoded by the *fim* operon, are commonly found in enterobacteria such as *E. coli* and are involved in the attachment of bacterial cells to the surface of specific receptors on eukaryotic cells, thus enabling adhesion [36]. Type 1 fimbriae contribute to the increased virulence of *E. coli* in the urinary tract, thereby promoting bacterial persistence [37]. The expression of type 1 fimbriae in the cellular envelope of uropathogenic *E. coli* allows attachment to the epithelium of the bladder and lower urinary tract, leading to colonization and the establishment of infection [38]. Type 1 fimbriae are also considered important virulence factors in *K. pneumoniae *, as they mediate the initial adhesion to host cells [39].

In this study, we analysed the isolates for the presence of two genes encoding the largest subunits of type 1 and 3 fimbriae: *fim*A and *mrk*A. In adhesion tests, type 1 fimbriae are inhibited by d-mannose; however, under these conditions, adhesion using other adhesins, such as type 3 fimbriae, can occur. Only 18 of the analysed isolates adhered to HeLa cells in an aggregative manner in the presence of d-mannose, whereas all isolates adhered without d-mannose. This could be explained by the inhibition of type 1 fimbriae by d-mannose.

Biofilm formation is vital for bacterial colonization at the start of an infection, whether or not the infection is associated with a device [40]. Mobley *et al*. [41] reported that *P. stuartii* isolates expressing MR/K fimbriae adhere to catheters more easily than isolates that do not express this type of adhesin. MR/K fimbriae are related to the persistence of a pathogen in the catheter as, after adhesion, a biofilm can form, which contributes to the establishment of an infection. The expression of type 3 fimbriae is essential for biofilm formation in various bacterial species [39].

Type 3 fimbriae may not be directly involved in urinary tract infections (UTIs) but may be essential in device-associated infections such as catheter-associated UTI. Thus, type 3 fimbriae-encoding genes such as *mrk*A play an essential role in biofilm formation in *K. pneumoniae *, which is important for initial cell-to-surface fixation and cell-cell adhesion [39].

*E. coli* haemolysin, encoded by the *hlyA* gene, is one of the main virulence factors and the best-studied haemolysin. HlyA is a member of a large family of exotoxins produced by several Gram-negative bacteria, including *Proteus* and *Morganella* spp. Haemolysin lyses erythrocytes via osmotic shock, resulting in hydrophilic pores on the cell surface [42]. *E. coli* haemolysin produces a clear zone around colonies on blood agar owing to erythrocyte lysis [43]. The ruptured erythrocytes release haemoglobin, and haemoglobin from lysed erythrocytes is an excellent source of iron because it contains four haem groups, each bound to an iron molecule released through cell lysis [44].

After haemoglobin is released from erythrocytes, bacterial cells must take up haem. Several pathogenic bacteria secrete molecules called siderophores, such as those encoded by the genes examined in this study, which bind to ferrous iron with high affinity and transport it back to the bacteria, or encode receptors that bind haem directly [44, 45].

Cytotoxicity analysis revealed that all the tested isolates showed some cytotoxicity against Vero cells, and 33/45 isolates were considered highly cytotoxic, killing 50 % or more of the cultured cells. Many factors such as the production of toxins and proteases can influence the cytotoxicity of pathogens. For example, although HlyA is cytolytic towards erythrocytes, it has also been reported to lyse other cell types in a variety of human and animal hosts [46].

At high concentrations, HlyA forms multimeric pores in eukaryotic membranes, leading to cell lysis, whereas at lower concentrations, it can interfere with host-cell signalling pathways and cause cell death by apoptosis [45]. HlyA from *E. coli* induces oxidative stress, leading to the accumulation of free radicals and cell death due to oxidative damage [47].

In the present study, all isolates had phenotypic characteristics of ESBL producers, and the investigation of β-lactamase genes by PCR revealed that all 45 isolates carried the *bla*_CTX-M-2_ gene and 23 isolates carried the *bla*_CTX-M-9_ gene, corroborating the study by Liu *et al*. [48], where all *P. stuartii* isolates tested were considered ESBL.

In a study by Zavascki *et al*. [49] carried out at a Brazilian university hospital, all analysed *P. stuartii* isolates were also positive for *bla*_CTX-M-2_; however, no other *bla*_CTX-M_ genes were found. The *bla*_CTX-M-2_, *bla*_CTX-M-8_ and *bla*_CTX-M-9_ genes are most commonly found in ESBL-producing enterobacteria in South American countries [50]. In Brazil, *bla*_CTX-M-2_ is one of the most prevalent genes, along with *bla*_CTX-M-15_, which occurs more frequently in the southeastern region [51].

Since the first description of *bla*_KPC_ genes, carbapenemase-producing *K. pneumoniae* isolates have become a major concern worldwide. Other enterobacteria carrying the *bla*_KPC-2_ gene have also been reported; however, *P. stuartii* isolates carrying this gene are uncommon. Isolates of *P. stuartii* carrying the *bla*_KPC-2_ gene have already been described by Tavares *et al*. [52] and Aires *et al*. [53], and the isolates carrying this gene were found in our study. This clearly demonstrates the need for the immediate recognition of these isolates, so that control measures can be established to prevent their spread within hospitals.

Based on the results presented in the dendrogram (Fig. 2), it is possible to state that there is similarity between the isolates analysed. However, we cannot conclude whether these isolates are clonally related or are the result of intrahospital transmission. Therefore, we suggest that future studies be conducted to elucidate this issue.

Some studies have reported clonal relationships among *P. stuartii* strains of clinical origin. In a study by Saida *et al*. [54], two clones of *P. stuartii* were shown to be disseminated among patients in a burn ward, and in a study by Douka *et al*. [55], a pan-resistant clonal strain of *P. stuartii* was shown to be the cause of infections in an intensive care unit. These studies indicate the possibility of the nosocomial dissemination of *P. stuartii *.

These pathogens can originate from the patient’s own microbiota and can be transferred to the site of infection. In addition, pathogens can be disseminated by the treatment team through direct contact with patients. Treatment teams can also transport pathogens to other environments and other patients [56].

Another alarming factor in outbreaks caused by *P. stuartii* is the resistance of these micro-organisms to commonly used antimicrobials, in addition to the resistance genes acquired by horizontal gene transfer. This highlights the clinical importance of *P. stuartii* as an emerging pathogen and the concerns regarding its ability to acquire and disseminate resistance genes [9].

## Conclusion

Based on our findings, it can be concluded that the *P. stuartii* isolates exhibit several virulence traits that make them highly successful pathogens. These include a strong capacity for adhesion to HeLa cells, cytotoxicity to Vero cells, haemolytic activity on blood agar, and the ability to form biofilms. Additionally, all isolates contained genes encoding various virulence factors, such as fimbriae, haemolysin, and iron and haem acquisition systems. In particular, all tested isolates were positive for ESBL and genes encoding beta-lactamases, indicating a high potential for antimicrobial resistance. This was further supported by the fact that all the isolates were resistant to many commonly used antimicrobials, with [(8/45) 17.77 %] isolates carrying the *bla*_KPC-2_ gene. These findings emphasize the need for ongoing efforts to prevent the spread of drug-resistant strains of *P. stuartii* in hospitals. Clonality was revealed among the isolates through ERIC-PCR typing, suggesting that a single isolate may be responsible for multiple infections in hospitalized patients. This highlights the importance of implementing effective infection control measures, promoting proper hygiene practices and continually monitoring the emergence of new strains of *P. stuartii* to prevent the nosocomial transmission of these pathogens.

## Supplementary Data

Supplementary material 1
