# Supplementary material for: Epidemiology and characterization of Providencia stuartii isolated from hospitalized patients in southern Brazil: a possible emerging pathogen
Source: Access Microbiol. 2023 Oct 18;5(10):000652.v4. doi: 10.1099/acmi.0.000652.v4 (PMC10634494; doi:10.1099/acmi.0.000652.v4)
Supplement: Supplementary material 1 [file acmi-5-652.v4-s001.pdf]

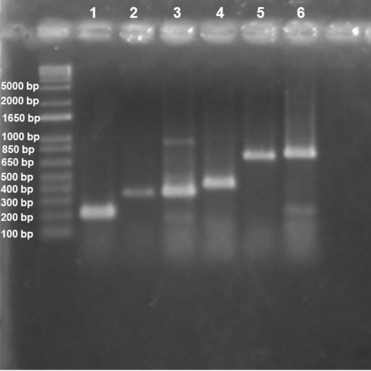

Supplementary figure 1 – Agarose gel containing positives samples for *P. stuartii* virulence primers.
Legend: 1: *fim*A gene (215 bp); 2: *iut*A gene (347 bp); 3: *ire*A gene (351 bp); 4: *mrk*A gene (411bp); 5: *hly*A gene (701 bp); 6: *fpt*A gene (744 bp).

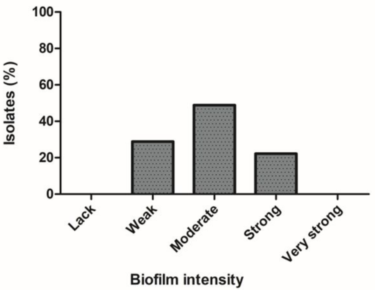

Supplementary figure 2 - Biofilm intensity of *P. stuartii* isolates

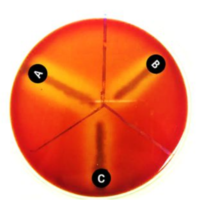

Supplementary figure 3 - Blood agar plate with hemolysis produced by *P. stuartii* isolates.
Legend: A: Isolate 1; B isolate 2; C: isolated 3.

**VFclass**	**Virulence factors**	**Related genes**	***Providencia stuartii* 33672(Prediction)**	***E. coli* CFT073(UPEC)**
			**NZ_CP008920 (NZ_CP008920)**	**chromosome (NC_004431)**
Adherence	AAF/II fimbriae	aafA	-	-
		aafB	-	-
		aafC	-	-
		aafD	-	-
	AAF/III fimbriae	agg3A	-	-
		agg3B	-	-
		agg3C	-	-
		agg3D	-	-
	Afimbrial adhesin AFA-I	afaA	-	-
		afaB	-	-
		afaC	-	-
		afaD	-	-
		afaE	-	-
		draP	-	-
	CFA/I fimbriae	cfaA	-	-
		cfaB	-	-
		cfaC	-	-
		cfaD/cfaE	-	-
	Curli fibers	cgsD	-	-
		cgsE	-	-
		cgsF	-	-
		cgsG	-	-
		csgA	-	-
		csgB	-	-
		csgC	-	-
	Dispersin	aap	-	-
	E. coli common pilus (ECP)	ecpA	-	c0404
		ecpB	-	c0403
		ecpC	-	c0402
		ecpD	-	c0401
		ecpE	-	c0400
		ecpR	-	c0405
	E.coli laminin-binding fimbriae (ELF)	elfA	-	-
		elfC	-	-
		elfD	-	-
		elfG	-	-
	EaeH	eaeH	-	c0415
	EtpA	etpA	-	-
	F1C fimbriae	focA	-	c1239
		focC	-	c1241
		focD	-	c1242
		focF	-	c1243
		focG	-	c1244
		focH	-	c1245
		focI	-	c1240

	Hemorrhagic E.coli pilus (HCP)	hcpA	-	c0127
		hcpB	-	c0126
		hcpC	-	c0125
	Intimin	eae	-	-
	K88 fimbriae	faeC	-	-
		faeD	-	-
		faeE	-	-
		faeF	-	-
		faeG	-	-
		faeH	-	-
		faeI	-	-
		faeJ	-	-
	P fimbriae	papA	-	c3592; c5188
		papB	-	c3592a; c5188a
		papC	DR96_RS03290; DR96_RS03805; DR96_RS07975; DR96_RS08505; DR96_RS09815; DR96_RS11505; DR96_RS12350	c3590; c5186
		papD	DR96_RS03800; DR96_RS08510; DR96_RS09820; DR96_RS11510; DR96_RS12355	c3589; c5185
		papE	-	c3585; c5181
		papF	-	c3584; c5180
		papG	-	c3583; c5179
		papH	-	c3591; c5187
		papI	-	c3593; c5189
		papJ	-	c3588; c5184
		papK	-	c3586; c5182
		papX	-	c3582
	Porcine attaching-effacing associated protein	paa	-	-
	S fimbriae	sfaA	-	-

		sfaB	-	-
		sfaC	-	-
		sfaD	-	-
		sfaE	-	-
		sfaF	-	-
		sfaG	-	-
		sfaH	-	-
		sfaS	-	-
	ToxB	toxB	-	-
	Type I fimbriae	fimA	DR96_RS03530	c5393
		fimB	DR96_RS12325	c5391
		fimC	DR96_RS03520	c5395
		fimD	DR96_RS00620; DR96_RS00990; DR96_RS01020; DR96_RS03515; DR96_RS06710; DR96_RS07770; DR96_RS09460; DR96_RS14870	c5396
		fimE	-	c5392
		fimF	DR96_RS03510	c5397
		fimG	DR96_RS03505	c5399
		fimH	DR96_RS03500	c5400
		fimI	DR96_RS03525	c5394
	Type 3 fimbriae(Klebsiella)		DR96_RS15020	-
			DR96_RS15015	-
		mrkA	DR96_RS15010	-
Autotransporter	AIDA-I type	tibA	-	-
	AIDA-I	aida	-	-
	AatA	aatA	-	-
	Antigen 43	agn43	-	c1273; c3655
	Cah	cah	-	-
	Contact-dependent inhibition CDI system	cdiA	-	c0345
		cdiB	-	-
	EhaA	ehaA	-	-
	EhaB	ehaB	-	-

	Enteroaggregative immunoglobulin repeat protein	air/eaeX	-	-
	EspC	espC	-	-
	EspI	espI	-	-
	EspP	espP	-	-
	Pet	pet	-	-
	Pic	pic	-	c0350
	Sat	sat	-	c3619
	Temperature-sensitive hemagglutinin	tsh	-	-
	UpaG adhesin	upaG/ehaG	-	c4424
	UpaH	upaH	-	-
	Vacuolating autotransporter gene	vat	-	c0393
Invasion	Invasion of brain endothelial cells (Ibes)	ibeA	-	-
		ibeB	-	c0658
		ibeC	DR96_RS10120	c4914
	Tia/Hek	tia	-	-
	Flagella(Burkholderia)	cheB	DR96_RS12925	-
		cheR	DR96_RS12920	-
Iron uptake	Aerobactin siderophore	iucA	-	c3627
		iucB	-	c3626
		iucC	-	c3625
		iucD	-	c3624
		iutA	DR96_RS02365	c3623
	Heme uptake	chuA	DR96_RS14065; DR96_RS14070	c4308
		chuS	DR96_RS14060	c4307
		chuT	DR96_RS15860	c4313
		chuU	DR96_RS14050	c4317
		chuW	DR96_RS15855	c4314
		chuX	DR96_RS15850	c4315
		chuY	DR96_RS15845	c4316
	Iron-regulated element	ireA	DR96_RS06490	c5174
	Iron/manganese transport	sitA	DR96_RS13895	c1600
		sitB	DR96_RS13890	c1599
		sitC	DR96_RS13885	c1598
		sitD	DR96_RS13880	c1597
	Salmochelin siderophore	iroB	-	c1254

		iroC	-	c1253
		iroD	-	c1252
		iroE	-	c1251
		iroN	-	c1250
	Yersiniabactin siderophore	fyuA	-	c2436
		irp1	-	c2429
		irp2	-	c2424
		ybtA	-	c2423
		ybtE	-	c2433
		ybtP	-	c2422
		ybtQ	-	c2421
		ybtS	-	c2419
		ybtT	-	c2432
		ybtU	-	c2430
		ybtX	-	c2420
	Heme transport(Shigella)	shuV	DR96_RS14045	-
	Pyochelin receptor(Pseudomonas)	fptA	DR96_RS09960	-
LEE-encoded TTSS effectors	EspB	espB	-	-
	EspF	espF	-	-
	EspG	espG	-	-
	EspH	espH	-	-
	Mitochondria-associated protein Map	map	-	-
	SepZ/EspZ	sepZ	-	-
	Tir	tir	-	-
Non-LEE encoded TTSS effectors	Cell-cycle-inhibitory factor Cif	cif	-	-
	EspFu/TccP (Tir cytoskeleton coupling protein)	espFu/tccP	-	-
	EspG2 (EPEC EspC island)	espG2	-	-
	EspJ	espJ	-	-
	EspK	espK	-	-
	EspL1	espL1	-	-
	EspL2	espL2	-	-
	EspL4	espL4	-	-
	EspM1	espM1	-	-
	EspM2	espM2	-	-
	EspN	espN	-	-
	EspO1-1	espO1-1	-	-
	EspO1-2	espO1-2	-	-
	EspR1	espR1	-	-
	EspR3	espR3	-	-
	EspR4	espR4	-	-
	EspV	espV	-	-
	EspW	espW	-	-
	EspX1	espX1	-	-
	EspX2	espX2	-	-
	EspX4	espX4	-	-
	EspX5	espX5	-	-
	EspX6	espX6	-	-
	EspX7	espX7	-	-

	EspY1	espY1	-	-
	EspY2	espY2	-	-
	EspY3	espY3	-	-
	EspY4	espY4	-	-
	EspY5	espY5	-	-
	LifA/Efa1	lifA/efa1	-	-
	NleA	nleA	-	-
	NleB1	nleB1	-	-
	NleB2-1	nleB2-1	-	-
	NleB2-2	nleB2-2	-	-
	NleC	nleC	-	-
	NleD	nleD	-	-
	NleE-1	nleE-1	-	-
	NleE-2	nleE-2	-	-
	NleF	nleF	-	-
	NleG-1	nleG-1	-	-
	NleG-2	nleG-2	-	-
	NleG-3	nleG-3	-	-
	NleG2-2	nleG2-2	-	-
	NleG2-3	nleG2-3	-	-
	NleG2-4	nleG2-4	-	-
	NleG5-1	nleG5-1	-	-
	NleG5-2	nleG5-2	-	-
	NleG6-1	nleG6-1	-	-
	NleG6-2	nleG6-2	-	-
	NleG6-3	nleG6-3	-	-
	NleG7	nleG7	-	-
	NleG8-2	nleG8-2	-	-
	NleH1-1	nleH1-1	-	-
	NleH1-2	nleH1-2	-	-
	TccP2	tccP2	-	-
Regulation	AggR	aggR	-	-
	PhoPQ(Salmonella)	phoQ	DR96_RS17335	-
Secretion system	AAI/SCI-II T6SS	aaiA	-	-
		aaiB	-	-
		aaiC/hcp	-	-
		aaiD	-	-
		aaiE	-	-
		aaiF	-	-
		aaiH	-	-
		aaiI	-	-
		aaiJ	-	-
		aaiK	-	-
		aaiL	-	-
		aaiM	-	-
		aaiN	-	-
		clpV/aaiP	-	-
		icmF/aaiO	-	-
		vgrG	-	-
	ABC transporter for dispersin	aatA	-	-
		aatB	-	-
		aatC	-	-

		aatD	-	-
		aatP	-	-
	ACE T6SS	Undetermined	-	-
		Undetermined	-	-
		aec11	-	-
		aec14	-	-
		aec15	-	-
		aec16	DR96_RS02005; DR96_RS05040	-
		aec17	DR96_RS06525	-
		aec18	-	-
		aec19	-	-
		aec22	-	-
		aec23	-	-
		aec24	-	-
		aec25	-	-
		aec26	DR96_RS06565	-
		aec27/clpV	-	-
		aec28	-	-
		aec29	-	-
		aec30	DR96_RS06590	-
		aec31	-	-
		aec32	DR96_RS03545	-
		aec7	-	-
		aec8	-	-
	LEE locus encoded TTSS	Undetermined	-	-
		Undetermined	-	-
		Undetermined	-	-
		Undetermined	-	-
		Undetermined	-	-
		cesD2	-	-
		cesD	-	-
		cesF	-	-
		cesT	-	-
		escC	-	-
		escD	-	-
		escF	-	-
		escI	-	-
		escJ	-	-
		escK	-	-
		escL	-	-
		escN	-	-

		escO	-	-
		escP	-	-
		escR	-	-
		escS	-	-
		escT	-	-
		escU	-	-
		escV	-	-
		espA	-	-
		espB	-	-
		espD	-	-
		etgA	-	-
		glrA	-	-
		glrR	-	-
		ler	-	-
		sepD	-	-
		sepL	-	-
		sepQ	-	-
	SCI-I T6SS	Undetermined	-	c3385
		Undetermined	-	c3386
		Undetermined	-	c3387
		Undetermined	-	c3388
		Undetermined	-	c3389
		Undetermined	-	c3391
		Undetermined	-	c3392
		Undetermined	-	-
		Undetermined	-	-
		Undetermined	-	c3393
		Undetermined	-	-
		Undetermined	-	-
		Undetermined	-	-
		Undetermined	-	c3396
		Undetermined	-	-
		Undetermined	-	c3398
		Undetermined	-	c3399
		Undetermined	-	-
		Undetermined	-	c3400
		Undetermined	-	-

		Undetermined	-	c3401
		Undetermined	-	c3402
		Undetermined	-	-
		Undetermined	-	-
		Undetermined	-	-
		Undetermined	-	-
		Undetermined	-	-
	Flagella (cluster I)(Yersinia)	flgB	DR96_RS12980	-
		flgC	DR96_RS12985	-
		flgF	DR96_RS13000	-
		flgH	DR96_RS13010	-
		flgJ	DR96_RS13020	-
		flhB	DR96_RS12945	-
		fliC	DR96_RS13190	-
		fliE	DR96_RS13160	-
		fliF	DR96_RS13155	-
		fliJ	DR96_RS13135	-
		fliN	DR96_RS13115	-
		fliR	DR96_RS13095	-
		fliS	DR96_RS13180	-
		fliZ	DR96_RS12755	-
	T6SS(Aeromonas)		DR96_RS06545	-
	Ysa TTSS(Yersinia)		DR96_RS06350	-
			DR96_RS06280	-
		acpY	DR96_RS06410	-
		sycB	DR96_RS06385	-
		ysaC	DR96_RS06325	-
		ysaE	DR96_RS06320	-
		ysaJ	DR96_RS06300	-

		ysaK	DR96_RS06340	-
		ysaN	DR96_RS06345	-
		ysaQ	DR96_RS06360	-
		ysaR	DR96_RS06365	-
		ysaS	DR96_RS06370	-
		ysaT	DR96_RS06375	-
		ysaU	DR96_RS06380	-
		ysaV	DR96_RS06335	-
		ysaW	DR96_RS06330	-
Toxin	Alpha-hemolysin	hlyA	-	c3570
		hlyB	-	c3573
		hlyC	-	c3569
		hlyD	-	c3574
	Colicin-like Usp	usp	-	-
	Cytolethal distending toxin	cdtA	-	-
		cdtB	-	-
		cdtC	-	-
	Cytotoxic necrotizing factor 1	cnf1	-	-
	Enterotoxin 1	set1A	-	-
		set1B	-	-
	Enterotoxin SenB/TieB	senB	-	-
	Heat-labile enterotoxin	eltA	-	-
		eltB	-	-
	Heat-stable enterotoxin 1 (EAST1)	astA	-	-
	Hemolysin/cytolysin A	hlyE/clyA	-	-
	Shiga-like toxin	stx1A	-	-
		stx1B	-	-
		stx2A	-	-
		stx2B	-	-
	Phytotoxin phaseolotoxin(Pseudomonas)	cysC1	DR96_RS01445	-
	RTX toxin(Vibrio)	rtxB	DR96_RS12060	-
		rtxD	DR96_RS12055	-
	The repeat in toxin (RTX)(Aeromonas)	rtxE	DR96_RS12050	-
Antiphagocytosis	Capsule(Klebsiella)		DR96_RS05255; DR96_RS05325; DR96_RS05330	-

Biofilm formation	AdeFGH efflux pump/transport autoinducer(Acinetobacter)	adeG	DR96_RS03690	-
Efflux pump	AcrAB(Klebsiella)		DR96_RS00850	-
	FarAB(Neisseria)	farB	DR96_RS06700	-
Endotoxin	LOS(Haemophilus)	htrB	DR96_RS12615	-
		lpxK	DR96_RS12115	-
		opsX/rfaC	DR96_RS05405	-
Immune evasion	Exopolysaccharide(Haemophilus)	galE	DR96_RS05340; DR96_RS19290	-
		galU	DR96_RS10000	-
Magnesium uptake	Mg2+ transport(Salmonella)	mgtB	DR96_RS12740	-
		mgtC	DR96_RS12735	-
Motility	Flagella(Bordetella)	motA	DR96_RS12890	-
		motB	DR96_RS12895	-
Others	MsbB2(Shigella)	msbB2	DR96_RS14850	-
	O-antigen(Yersinia)		DR96_RS04505	-
		cpsB	DR96_RS05310	-
Serum resistance	LPS rfb locus(Klebsiella)		DR96_RS05580	-
Stress adaptation	Catalase(Neisseria)	katA	DR96_RS18125	-
	SodCI(Salmonella)	sodCI	DR96_RS16240	-

Supplementary Table 1 – Comparison of the genome of *P. stuartii* 33672 with *E.coli* (UPEC) CFT073.

**VFclass**	**Virulence factors**	**Related genes**	***Providencia stuartii* 33672(Prediction)**	**K.*pneumoniae* KCTC 2242**	
			**NZ_CP008920 (NZ_CP008920)**	**chromosome (NC_017540)**	**pKCTC2242 (NC_017541)**
Adherence	Type 3 fimbriae	mrkA	DR96_RS15010	KPN2242_19355	-
		mrkB	DR96_RS15015	KPN2242_19350	-
		mrkC	DR96_RS06710; DR96_RS09460; DR96_RS15020	KPN2242_19345	-
		mrkD	-	KPN2242_19340	-
		mrkF	-	KPN2242_19335	-
		mrkH	-	KPN2242_19320	-
		mrkI	-	KPN2242_19325	-
		mrkJ	-	KPN2242_19330	-
	Type I fimbriae	fimA	DR96_RS03530	KPN2242_19395	-
		fimB	DR96_RS12325	KPN2242_19385	-
		fimC	DR96_RS03520	KPN2242_19405	-
		fimD	DR96_RS00620; DR96_RS00990; DR96_RS01020; DR96_RS03515; DR96_RS07770; DR96_RS14870	KPN2242_19410	-
		fimE	-	KPN2242_19390	-
		fimF	DR96_RS03510	KPN2242_19415	-

		fimG	DR96_RS03505	KPN2242_19420	-
		fimH	DR96_RS03500	KPN2242_19425	-
		fimI	DR96_RS03525	KPN2242_19400	-
		fimK	-	KPN2242_19430	-
	P fimbriae(Escherichia)	papD	DR96_RS03800; DR96_RS08510	-	-
Antiphagocytosis	Capsule	-	DR96_RS05255; DR96_RS05260; DR96_RS05265; DR96_RS05310; DR96_RS05320; DR96_RS05325; DR96_RS05330; DR96_RS05575; DR96_RS10000; DR96_RS11705	KPN2242_15535; KPN2242_15540; KPN2242_15545; KPN2242_15550; KPN2242_15565; KPN2242_15580; KPN2242_15585; KPN2242_15590; KPN2242_15595; KPN2242_15600; KPN2242_15605; KPN2242_15610; KPN2242_15615; KPN2242_15620; KPN2242_15625; KPN2242_15630	-
Efflux pump	AcrAB	acrA	DR96_RS00850	KPN2242_04625	-
		acrB	DR96_RS00855; DR96_RS07920	KPN2242_04620	-

	FarAB(Neisseria)	farB	DR96_RS06700	-	-
Iron uptake	Aerobactin	iucA	-	-	KPN2242_25281
		iucB	-	-	KPN2242_25276
		iucC	-	-	KPN2242_25271
		iucD	-	-	KPN2242_25266
		iutA	DR96_RS02365	KPN2242_08605	KPN2242_25261
	Ent siderophore	entA	-	KPN2242_05930	-
		entB	-	KPN2242_05925	-
		entC	-	KPN2242_05915	-
		entD	-	KPN2242_05865	-
		entE	-	KPN2242_05920	-
		entF	-	KPN2242_05885	-
		entS	-	KPN2242_05905	-
		fepA	-	KPN2242_05870	-
		fepB	-	KPN2242_05910	-
		fepC	-	KPN2242_05890	-
		fepD	-	KPN2242_05900	-
		fepG	-	KPN2242_05895	-
		fes	-	KPN2242_05875	-
	Salmochelin	IroB	-	-	-
		iroC	-	-	-
		iroD	-	-	-
		iroE	-	KPN2242_11295	-
		iroN	-	KPN2242_09340	-
	Yersiniabactin	fyuA	-	-	-
		irp1	-	-	-
		irp2	-	-	-
		ybtA	-	-	-
		ybtE	-	-	-

		ybtP	-	-	-
		ybtQ	-	-	-
		ybtS	-	-	-
		ybtT	-	-	-
		ybtU	-	-	-
		ybtX	-	-	-
	Ferrous iron transport(Shigella)	sitA	DR96_RS13895	-	-
	Heme transport(Shigella)	shuV	DR96_RS14045	-	-
	Heme uptake(Escherichia)	chuA	DR96_RS14065	-	-
		chuS	DR96_RS14060	-	-
		chuT	DR96_RS15860	-	-
		chuU	DR96_RS14050	-	-
		chuW	DR96_RS15855	-	-
		chuX	DR96_RS15850	-	-
		chuY	DR96_RS15845	-	-
	Iron-regulated element(Escherichia)	ireA	DR96_RS06490	-	-
	Iron/manganese transport(Escherichia)	sitB	DR96_RS13890	-	-
		sitC	DR96_RS13885	-	-
		sitD	DR96_RS13880	-	-
	Pyochelin receptor(Pseudomonas)	fptA	DR96_RS09960	-	-
Nutritional factor	Allantoin utilization	allA	-	-	-
		allB	-	-	-
		allC	-	-	-
		allD	-	-	-
		allR	-	-	-
		allS	-	-	-
Regulation	RcsAB	rcsA	-	KPN2242_15070	-
		rcsB	DR96_RS18075	KPN2242_16255	-
	RmpA	rmpA	-	-	KPN2242_26191
	PhoPQ(Salmonella)	phoQ	DR96_RS17335	-	-

Secretion system	T6SS-I	-	-	KPN2242_09790	-
		-	DR96_RS11060	KPN2242_09815	-
		-	-	KPN2242_09820	-
		clpV/tssH	-	KPN2242_09780	-
		dotU/tssL	-	KPN2242_09765	-
		hcp/tssD	-	KPN2242_09775	-
		icmF/tssM	-	KPN2242_09825	-
		impA/tssA	-	KPN2242_09830	-
		ompA	-	KPN2242_09770	-
		sciN/tssJ	-	KPN2242_09845	-
		tle1	-	KPN2242_09810	-
		tli1	-	KPN2242_09795; KPN2242_09800; KPN2242_09805	-
		tssF	-	KPN2242_09835	-
		tssG	-	KPN2242_09840	-
		vasE/tssK	-	KPN2242_09760	-
		vgrG/tssI	-	KPN2242_09785	-
		vipA/tssB	-	KPN2242_09750	-
		vipB/tssC	-	KPN2242_09755	-
	T6SS-II	clpV	DR96_RS10650	-	-
		dotU	-	-	-
		icmF	-	-	-
		impF	-	-	-
		impH	-	-	-
		impJ	-	-	-
		ompA	-	-	-
		sciN	-	-	-
		vasA/impG	-	-	-

		vgrG	-	-	-
	T6SS-III	-	-	-	-
		-	-	-	-
		-	-	KPN2242_14290	-
		-	-	-	-
		-	-	KPN2242_14270	-
		-	-	KPN2242_14235	-
		-	-	KPN2242_14230	-
		dotU	-	KPN2242_14315	-
		icmF	-	KPN2242_14265	-
		impA	-	KPN2242_14240	-
		impF	-	KPN2242_14245	-
		impG	-	KPN2242_14260	-
		impH	-	KPN2242_14255	-
		impJ	-	KPN2242_14320	-
		lysM	-	-	-
		ompA	-	KPN2242_14310	-
		sciN	-	KPN2242_14250	-
		vgrG	-	KPN2242_14300	-
	ACE T6SS(Escherichia)	aec26	DR96_RS06565	-	-
		aec30	DR96_RS06590	-	-
		aec32	DR96_RS03545	-	-
	Flagella (cluster I)(Yersinia)	flgB	DR96_RS12980	-	-
		flgC	DR96_RS12985	-	-
		flgF	DR96_RS13000	-	-
		flgG	DR96_RS13005	-	-
		flgH	DR96_RS13010	-	-
		flgI	DR96_RS13015	-	-

		flgJ	DR96_RS13020	-	-
		flhB	DR96_RS12945	-	-
		flhC	DR96_RS12885	-	-
		flhD	DR96_RS12880	-	-
		fliA	DR96_RS13195	-	-
		fliC	DR96_RS13190	-	-
		fliE	DR96_RS13160	-	-
		fliF	DR96_RS13155	-	-
		fliG	DR96_RS13150	-	-
		fliI	DR96_RS13140	-	-
		fliJ	DR96_RS13135	-	-
		fliM	DR96_RS13120	-	-
		fliN	DR96_RS13115	-	-
		fliP	DR96_RS13105	-	-
		fliQ	DR96_RS13100	-	-
		fliR	DR96_RS13095	-	-
		fliS	DR96_RS13180	-	-
		fliZ	DR96_RS12755	-	-
	T6SS(Aeromonas)		DR96_RS06525	-	-
			DR96_RS06545	-	-
		hcp	DR96_RS02005; DR96_RS05040	-	-
	Ysa TTSS(Yersinia)		DR96_RS06350	-	-
			DR96_RS06280	-	-
		acpY	DR96_RS06410	-	-

		sycB	DR96_RS06385	-	-
		ysaC	DR96_RS06325	-	-
		ysaE	DR96_RS06320	-	-
		ysaJ	DR96_RS06300	-	-
		ysaK	DR96_RS06340	-	-
		ysaN	DR96_RS06345	-	-
		ysaQ	DR96_RS06360	-	-
		ysaR	DR96_RS06365	-	-
		ysaS	DR96_RS06370	-	-
		ysaT	DR96_RS06375	-	-
		ysaU	DR96_RS06380	-	-
		ysaV	DR96_RS06335	-	-
		ysaW	DR96_RS06330	-	-
Serum resistance	LPS rfb locus	-	DR96_RS05580	KPN2242_15475; KPN2242_15480; KPN2242_15485; KPN2242_15490; KPN2242_15495; KPN2242_15505; KPN2242_15510; KPN2242_15515; KPN2242_15520; KPN2242_15525	-
Toxin	Colibactin	clbA	-	-	-
		clbB	-	-	-
		clbC	-	-	-
		clbD	-	-	-
		clbE	-	-	-

		clbF	-	-	-
		clbG	-	-	-
		clbH	-	-	-
		clbI	-	-	-
		clbJ	-	-	-
		clbK	-	-	-
		clbL	-	-	-
		clbM	-	-	-
		clbN	-	-	-
		clbO	-	-	-
		clbP	-	-	-
		clbQ	-	-	-
		clbS	-	-	-
	Phytotoxin phaseolotoxin(Pseudomonas)	cysC1	DR96_RS01445	-	-
	RTX toxin(Vibrio)	rtxB	DR96_RS12060	-	-
		rtxD	DR96_RS12055	-	-
	The repeat in toxin (RTX)(Aeromonas)	rtxE	DR96_RS12050	-	-
Biofilm formation	AdeFGH efflux pump/transport autoinducer(Acinetobacter)	adeG	DR96_RS03690	-	-
Endotoxin	LOS(Haemophilus)	htrB	DR96_RS12615	-	-
		lpxK	DR96_RS12115	-	-
Fimbrial adherence determinants	Stf(Salmonella)	stfD	DR96_RS09820; DR96_RS11510; DR96_RS12355	-	-
Immune evasion	Exopolysaccharide(Haemophilus)	galE	DR96_RS05340; DR96_RS19290	-	-
Invasion	Flagella(Burkholderia)	cheB	DR96_RS12925	-	-
		cheR	DR96_RS12920	-	-
		cheW	DR96_RS12905	-	-
		cheY	DR96_RS12930	-	-
	Invasion of brain endothelial cells (Ibes)(Escherichia)	ibeC	DR96_RS10120	-	-

Magnesium uptake	Mg2+ transport(Salmonella)	mgtB	DR96_RS127 40	-	-
		mgtC	DR96_RS127 35	-	-
Motility	Flagella(Bordetella)	motA	DR96_RS128 90	-	-
		motB	DR96_RS128 95	-	-
Others	MsbB2(Shigella)	msbB2	DR96_RS148 50	-	-
	O-antigen(Yersinia)		DR96_RS045 05	-	-
Stress adaptation	Catalase(Neisseria)	katA	DR96_RS181 25	-	-
	SodCI(Salmonella)	sodCI	DR96_RS162 40	-	-

Supplementary table 2 – Comparison of the genome of *P. stuartii* 33672 with *K. pneumonieae* KCTC 2242.
